# Perspectives regarding privacy in clinical research among research professionals from the Arab region: an exploratory qualitative study

**DOI:** 10.1186/s12910-020-0456-9

**Published:** 2020-04-15

**Authors:** Latifa Adarmouch, Marwan Felaefel, Robert Wachbroit, Henry Silverman

**Affiliations:** 1 Cadi Ayyad University School of Medicine Community Medicine and Public Health Department, Marrakech, Morocco; 2 The American University in Cairo, Cairo, Egypt; 3 University of Maryland School of Medicine, Baltimore, MD USA

**Keywords:** Privacy, Confidentiality, Clinical research, Egypt, Morocco

## Abstract

**Background:**

Protecting the privacy of research participants is widely recognized as one of the standard ethical requirements for clinical research. It is unknown, however, how research professionals regard concepts of privacy as well as the situations in the research setting that require privacy protections. The aim of this study was to explore the views of research professionals from Arab countries regarding concepts and scope of privacy that occur in clinical research.

**Methods:**

We adopted an exploratory qualitative approach by the use of focus group discussions. We recruited individuals involved in research from Egypt and Morocco. We analyzed focus group data via a constant comparison approach, which consisted of close reading of the transcribed interviews followed by coding and then determining themes and subthemes.

**Results:**

Between August 2016 and July 2018, we conducted nine focus group discussions. Respondents discussed several privacy issues that occurred before the research began (e.g., recruitment practices); during research (e.g., data collection and physical exams), and after the research (e.g., secondary use of data and data sharing). Respondents revealed their perspectives of patients towards privacy in the clinical and research settings and mentioned that patients are more likely to permit access to their privacy in the clinical setting compared with research setting due to the existence of benefits and trust in clinical care. Respondents also recommended training regarding data protections for individuals involved in research.

**Conclusions:**

Our study shows that research professionals discussed a range of privacy issues that are present during the different stages of research. We recommend 1) development of standards regarding privacy protections during recruitment efforts; 2) additional training for individuals involved in research regarding best practices with data security in secondary research; 3) a quantitative study involving investigators and REC members to determine their knowledge, attitudes and practices regarding privacy issues that occur in research; and 4) a quantitative study involving patients to elicit their views regarding their privacy concerns in research.

## Background

Protecting the privacy of research participants is recognized as one of the standard ethical requirements for clinical research. An immediate challenge to any study of privacy protection is that there is no consensus amongst lawyers, policymakers, health professionals, and philosophers on the definition of privacy. Indeed, the literature shows that privacy does not refer to a single concept but rather to a cluster of concepts [1, 2]. Although there are multiple definitions of privacy, it still remains “A concept in disarray” [3]. For example, Westin describes privacy in terms of information control: “the claim of individuals…to determine for themselves when, how, and to what extent information about them is communicated to others” [4]. Inness defines privacy as “the state of possessing control over a realm of intimate decisions, which include decisions about intimate access, intimate information, and intimate actions [5]. Moore highlights having “control” over access as well as “use” of any information obtained, as yielding control over access does not necessarily yield control over use [1]. Marmor contends that the “right to privacy…is there to protect our interest in having a reasonable measure of control over ways in which we present ourselves to others….and the ability to present different aspects of ourselves. to different people”, which is an important aspect of our well-being. In addition to control of information, others have brought up the importance of physical privacy that includes notions of modesty, intimacy, and bodily integrity [6].

A related challenge is that privacy protections in research might not be the same as privacy protections in clinical care. Furthermore, unfamiliarity with concepts of privacy might lead to a failure to appreciate the broad scope of privacy, a state of affairs that might lead to insufficient privacy protections for research participants. These considerations and their impact on the privacy protection requirements in the research setting in the Arab region have not been given much attention in the scholarly literature. Indeed, scholars have documented the paucity of publications on privacy and confidentiality in the Middle East compared with other world regions [7].

A sole focus on informational privacy, which is perhaps exacerbated by the belief that all that privacy in research requires is a focus on confidentiality assurances of research participants’ data, ignores other types of privacy, such as physical privacy, proprietary privacy, decisional privacy, and association privacy [6, 8]. Physical privacy involves the protection of individuals’ physical (bodily modesty and integrity) and personal space (solitude); proprietary privacy includes control over personal identify and likeness, which includes control over photographs, facial features, and genetic information (controlling the “fate of the building blocks of life” [6]; decisional privacy has been labeled as a type of freedom and includes the ability to make choices without the interference from others, (e.g., government) as well as freedom from intrusions and interference of the mind that can manipulate decisions and behavior; e.g., hyper nudging that occurs on social media [9]; and association privacy includes the ability to congregate in groups without interference. While informational privacy in research is certainly important, as the collection of private, sensitive data is what enables the aim of research, but activities involved with recruitment and the collection of data include intrusions in the other spheres of privacy (e.g., physical, proprietary, and decisional) to which permission is required from the participants.

A predominant focus on informational privacy was demonstrated by a recent qualitative study of Reasearch Ethics Committes (REC) members’ understanding of privacy and challenges to privacy [10], as well as in articles that emphasize the importance of data security in clinical trials — an investigation by Tucker et.al. serves as an example [11]. In a recent integrative literature review to examine topical aspects of privacy in research practice, the authors focus was mainly on informational privacy [12]. While the authors recognized the different concepts of privacy, they stated: “in a clinical-research context, the focus is typically on informational privacy” [12].

Research ethics guidelines also demonstrate a dominance on informational privacy at the exclusion of the other types of privacy . For example, The Guideline for Good Clinical Practice focus only on informational privacy as it states: “The confidentiality of records that could identify subjects should be protected, respecting the privacy and confidentiality rules…” [13]. A similar solitary focus on informational privacy is exemplified by the following statement in India’s regulations: “Principle of ensuring privacy and confidentiality whereby to maintain privacy of the potential participant, her/his identity and records are kept confidential and access is limited to only those authorized [14]. The Declaration of Helsinki states: “Every precaution must be taken to protect the privacy of research subjects and the confidentiality of their personal information” [15]. The Council for International Organizations of Medical Sciences (CIOMS) regulations states: “Every precaution must be taken to protect the privacy of research subjects and the confidentiality of their personal information” [16]. While it can be construed correctly that both of these latter documents distinguish confidentiality from the more genera term of ‘privacy’, they do not unpack the concept of privacy. Specifically, these guidelines leave the reader with the impression that protection of privacy encompasses solely efforts at data security.

The Canada’s TriCouncil Policy Statement conveys a broad definition of privacy when it refers to privacy as “an individual’s right to be free from intrusion or interference by others….Individuals have privacy interests in relation to their bodies, personal information, expressed thoughts and opinions, personal communications with others and spaces they occupy. Research affects these various domains of privacy in different ways depending on its objectives and method….” [17]. However, the document states later that “Privacy risks in research relate to the identifiability of participants…” thus emphasizing protections only in the realm of informational privacy, i.e., data security [17].

Morocco recently passed legislation, the Protection of Persons Participating in biomedical research, which states “The privacy of the participant and the confidentiality of the data concerning him must be respected….” [18]. But in Article 3 regarding the principles with which biomedical research must comply, it merely emphasizes “respect for the confidentiality of personal data”. Egypt’s draft clinical trial is still being discussed in parliament, but a current draft mentions respect for the “confidentiality of the data” without any reference to other privacy concerns (personal communication, anonymous). Hence, similar to the international guidelines, specification of other privacy protections is not mentioned in these national documents.

In contrast, the Constitutions of Egypt and Morocco specify individuals’ right to privacy that extends beyond informational privacy. For example, the Egyptian Constitution of 2014 contains an explicit protection of the right to privacy: Article 57 states “Private life is inviolable, safeguarded and may not be infringed upon and Article 99 states that “…any assault on…the inviolability of citizens’ private lives…shall be considered a crime” [19]. Similarly, the Moroccan constitution states: “The physical or moral integrity of anyone may not be infringed….” [20]. While the concept of privacy is given recognition in these constitutions, further specification of the scope of privacy is missing.

With this background in mind, we designed this study to explore the views of research professionals from Arab countries; specifically, investigators, clinical research associates, and members of RECs regarding their concepts about privacy in the research setting.

## Methods

### Study design

We adopted an exploratory qualitative approach by the use of focus group discussions (FGDs). The FGDs were conducted by two of the authors, one from Morocco (LA) and the other from Egypt (MF). We developed a semi-structured interview guide that consisted of questions related to participants’ knowledge and perceptions towards concepts of privacy in daily life as well as in the clinical and research settings (see Additional file 1: Interview guide). Specifically, the guide included the following questions:

- What are conceptions of privacy in daily life and why is it important?
- What are expectations of privacy in healthcare?
- What are expectations of privacy in clinical research?
- Please give examples of privacy situations before the research begins, during research, and after research.

Our initial questions were designed for respondents to initially discuss among themselves the meaning and importance of privacy in daily life and in the healthcare setting in order for them to reflect on the general issue of privacy before they discussed aspects of privacy in research. The interview guide was continually revised to explore the additional concepts that emerged from the focus groups.

Participants gave verbal informed consent, which was recorded in the beginning of the focus group discussion. Participants used different names to protect their privacy. The focus groups were conducted either in English, French or spoken Arabic and were audio recorded. The recordings were translated to English (when needed) and then transcribed. The actual number of FGDs was determined when “saturation” was reached in the data collected (i.e., redundancy of responses was identified).

### Recruitment

We aimed to recruit a broad range of individuals involved in research, which included junior investigators, medical faculty, members of RECs, and clinical research associates. Participants were from Egypt and Morocco. Each of the two interviewers (authors LA and MF) obtained a convenient study sample using their professional network. Potential participants were approached through an email or phone call and those who expressed interest were provided with detailed information about the study and their potential participation in writting. Individuals who agreed to participate were invited to a focus group discussion.

### Analysis

We analyzed focus group data via a constant comparison approach [21]. Analysis of the interviews proceeded through a series of sequential steps: close reading of the transcribed interviews followed by open-coding in which the data were chunked into small units. Codes were attached to these data chunks and organized by using Nvivo software version 11 for Mac. Codes were derived both deductively (based on the themes constituting the interview guide) and inductively (allowing for new codes to be considered from the text). The developed codes were then grouped into categories followed by development of themes. This process was then verified by the other author (HS); variability was discussed with authors LA and MF until consensus was reached. Themes were finalized by several iterative discussions between the authors.

### Ethics

The study protocol was reviewed and approved by the ethical review boards in Morocco (Comite D’Ethique Pour La Reseherche Biomedicale, Casablanca), Egypt (National Hepatology and Tropical Medicine Research Institute, Cairo, Egypt), and the USA (University of Maryland School of Medicine).

## Results

Between August 2016 and July 2018, nine FGDs were conducted. There were four FGDs from Morocco and five from Egypt. Demographic information about the participants of the FGs as well as number of participants of each focus group and duration of the interviews are presented in Table 1. Participants of the focus groups represented a diverse population that included junior investigators, medical faculty, members of research ethics committees, and clinical research associates. The identified themes and subthemes are shown in Table 2.

**Table 1:** Profile of Focus Groups

Number	Country	Profile of focus group	Number of participants	Males	Females	Duration of interview
1	Egypt	RECs/Medical faculty	5	2	3	1:15
2	Egypt	Clinical Research Associates	6	2	4	2:39
3	Egypt	Clinical Research Associates	4	1	3	1:45
4	Egypt	Medical faculty	7	2	5	1:25
5	Egypt	RECs/Medical faculty	5	1	4	1:20
6	Morocco	Junior Investigators	5	4	1	1:25
7	Morocco	Medical faculty	6	3	3	1:28
8	Morocco	Medical faculty	9	2	7	1:40
9	Morocco	Medical faculty	6	3	3	1:30

**Table 2:** Themes and Subthemes

1. Concepts of Privacy	
Definition of Privacy • Privacy as control of access • Privacy as a right • Awareness of privacy	
Scope of privacy • Information: demographic, religion, political • Physical (bodily) • Proprietary • Association	
Importance of Privacy • Intrinsic • Instrumental	
Importance of culture on scope of privacy	
2. Perspectives on privacy concerns of patients in the research setting	
Privacy in clinical care compared with research • Expectations of privacy are greater in the clinical setting compared with the research setting • Expectations of privacy are greater in the private setting compared with public setting • Reasons why individuals permit greater access to privacy in the clinical setting compared to research setting o Benefits are perceived to be greater in clinical care o Greater trust in clinicians compared with researchers • Knowledge of the aims of research can enhance potential research participants’ willingness to allow access to their privacy	
3. Privacy issues during different stages of research	
Privacy issues before research is conducted • Recruitment process • Informed consent setting	
Privacy issues during research • Third parties having access to private information • Gender Issues that occur with data collectors and indivduals who perform physical exams • Observation research	
Privacy issues after research • Use of data and samples in secondary research – informed consent issues • Data sharing • Genetic research and return of results	
4. Clarification of standards regarding privacy in research	
• Reconciliation of different views regarding data security in secondary research. • The need for training regarding privacy laws	

### Definition of Privacy

Several participants defined privacy in terms of control of who can access their “information” and “physical space”. One participant gave an example of such control in the realm of social media:

“ if I'm every five minutes posting a status update or posting a photo on Facebook to all the people in my friends’ feed or publicly or whatever, it's my choice to share this part of my life with the people who will see it, whether it's friends…or followers or whatever. It's good. However, if I choose not to post anything on Facebook, it's because I don't want to share the information that's happening with me right now with the friends or publicly.

### Privacy as a right

Several respondents used the language of rights when discussing privacy and included the following:

“... we have a right to define the terms of our privacy in relation to our preferences and our right to choose.”

“if you don’t want to answer any question, it is your right… he is participating in this research, but if he feels he don’t want to talk about something, it is his right.”

Another respondent brought up the ethical and religious foundations of privacy:

“The Hippocratic oath, which stipulates that we must commit to observing patients’ privacy... at all costs… In Arabic, it is even more stringent because one has to swear to God to respect the patients’ privacy, this becomes a major responsibility for us.”

### Awareness of Privacy

Several respondents doubted whether individuals are aware of their rights to privacy as exemplified by the following statements:

“Moroccans are not aware of their right to privacy and thus they readily divulge information and do not establish physical privacy limits.”

“Privacy itself is not a well-known concept here in Egypt….everyone can share their information without asking permission… so privacy…is actually not an established concept...”

“but because we are well-educated, we understand what privacy is…But most of the community doesn’t think of privacy at all. It’s none of their concern, seriously…. They don’t know that there are laws…They don’t know their rights about privacy….Do they know that doctors are sharing their information with other people.”

### The importance of Privacy

Respondents discussed the importance of privacy as a prerequisite for individual autonomy and is a constitutive element of their flourishing as unique individuals. The instrumental value of privacy was discussed as a means to keep disruptive material out of the “crippling” effects of the public “gaze”. For example, one participant said:

“For me it’s a form of protection, in other words, we protect the things that belong to us and are constantly afraid of being judged or of resulting repercussions.”

“I see this privacy thing is related more to this culture of “bad eye” and envy, people shouldn’t know, if they know I will not achieve my goals.”

Respondents gave specific examples of the “crippling” effects of the public “gaze” to include stigmatization and discrimination in regard to employment as several mentioned the following:

“And some people become less employable if their health-related issues become public.” And it does impact more than just their income or their value to society or whatever. It's very psychological.”

### Scope of Privacy

Respondents remarked on several types of privacy. When discussing informational privacy, respondents mentioned the following types of information that should be considered private:

- Demographics: age, gender, employment status, financial aspects, and marital status
- Social information: religious beliefs, sexual orientation, addictions.
- Health information: illnesses (e.g., HCV, HIV, cancer, sexual dysfunction), medications
- Genetic testing results
- Research participation

Other respondents brought up physical privacy; for example:

“There is the body privacy...people don’t want to be naked in front of another one, especially for women in front of male doctors and even males in front of female doctors.”

“Sometimes a patient can have a nice feature that is fake; dental bridges and fillings for example, people do not want others to know that they are fake. They want others to think they are natural, because sometimes others could pick on the patient for installing a fake thing.”

Several respondents alluded to proprietary privacy in regard to their pictures: “I, for one, consider a person’s personal stuff, such as his I.D and pictures, as part of his privacy”. Respondents also referred to association privacy in the political context, for example: “Depending on who you hang out with, you may want to keep that private.”

### Influence of culture on the scope of privacy

Participants emphasized how the scope of privacy can be influenced by culture. For example, the stigmatization that results from a violation of privacy is dependent on what is considered taboo in an individual culture. One respondent mentioned the following::

“Like being a smoker, or a wine drinker, which is not really socially accepted, ... Especially women who smoke, because many people find that unacceptable.”

Another respondent brought up individuals’ preference to be cared for by a healthcare provider of the same gender:

“ I mean everywhere women would like to be examined by a lady for example. I don’t think it’s because of the trust of the other lady. But I think it is probably culture, probably her beliefs, probably her perception.”

Respondents discussed the differences in cultures between rural and urban contexts with regard to privacy:

“In villages, privacy is regarded as a very important thing, meanwhile it might not be considered as such in cities…. The privacy of women in villages for instance. Consider how men refer to their wives by saying “the Mrs.” rather than revealing their real names in public. This is…culture and it has nothing to do with religion. It is observed by both, Muslims and Christians, living in rural villages…You will not find that in cities, of course.

“For me as a gynecologist physical privacy varies widely more so with residents from remote areas who...have great difficulty revealing themselves and being more open with their physical privacy. On the other hand, those who come from areas where the culture is a bit more open-minded, tend to be…less restrictive with their physical privacy.”

### Difference in the expectations of privacy between the medical care and research settings

Respondents mentioned that patients’ expectations of privacy are less in the research setting compared with the medical setting. Participants mentioned the following:

“The patient thinks that being investigated [means that] others would have access to their personal information.”

“They could also be anxious [because they think] that all the information contained in their files are going to be revealed.”

“In some researchers, like dermatological ones, pictures and even videos are taken. Here the patient really starts worrying about his privacy and whether or not it will be maintained”.

“In some consents there are items stating that some scientific authorities will ask for access to your information and thus the information have to be provided. These exceptions put the patient under stress as his privacy might be threatened.”

“So, in general, everyone thinks that sharing information for research is less than what you share for medical treatment”

Respondents discussed the importance of trust in the clinical setting compared with the research setting as reasons for the differences in the expectations of privacy between the two settings: The following responses were obtained:

“In a clinical setting a person may reveal many things, more so than in the context of research because they trust their doctor, there is only one person asking questions and they know that it is confidential…”

“Actually, in research, it is more difficult when it comes to privacy. The medical privacy between the doctor and his patient is a very strong relationship between both parties. However, in case of research, …he usually asks for more guarantees that his privacy [will be maintained].

Respondents also mentioned their perspectives that the willingness of patients to give permission to access their information depends on whether they expect to receive medical benefit.

“In healthcare if he comes to be treated, he has the impression that he will get a direct benefit from telling the needed information…. But in the procedure of scientific research he would say: “what I am going to win from this?”. “If I share information, in the health-care, it will help to have an exact diagnosis”. However, in scientific research, he can’t see a direct benefit from the questionnaire.”

Respondents believed that even in the context of research with the prospect of direct benefit (e.g., drug clinical trial), individuals need to believe that they will receive benefit.

“The patient must be confident in the outcome of the research and the doctor conducting the research….If the patient believes in the research, he will disclose anything… Understand that he suffers from a disease that many others also suffer from and that they might be able to reach a better state than the one he reached.

Respondents discussed that the inclination to reveal private information in research can be strengthened if participants understood better the underlying aims of research. Respondents said the following:

“For me the culture of scientific research is not well understood…our culture doesn’t include anything called scientific research”.

“But one of the main obstacles for me in years of conducting research whether clinical or trial or something like that, is the lack of awareness or knowledge… about the benefit that it is needed for the benefit of other society or anyone. So, it is mainly about the awareness of the people about the field itself.”

“…usually he doesn’t see the benefit for public health.

“Can we say that the same thing applies when the patient is part of a research rather than a treatment? Does the patient display the same willingness to disclose information for the sake of a research and not a treatment?”

### Privacy issues before the research begins

Respondents commented on privacy issues that occur before the research begins. One issue involved the method of recruitment and included the appropriate accesss to patients’ databases to identify those who meet inclusion/exclusion criteria. One respondent said the following:

“for example, you have a database and a pharma company wants to recruit from your patient’s database; are you going to share the database with the research team? Does this jeopardize the patient’s right to privacy? In that case you are obliged to seek the patient’s consent before.”

There were opposing viewpoints regarding the appropriateness of investigators searching the hospital databases for patients. One exchange between respondents was as follows:

F(1): The PI calls the patients? Without referring to the original doctor?

M(1): It's fine because he works at the hospital.

F(2): Yeah, the patient knows that his data are in the system in the hospital, so if anyone calls from the hospital, it's still fine, as long as it's within the same system because the patient is leaving his records in there.

Essentially, respondents believed investigators from the same institute had the right to access the data of any patient in the institute. However, other respondents thought that the non-treating doctor should not have direct access to patients’ list and offered an alternative process:

M(1): What the doctor can do is advise the patient, ‘Listen, there is a trial going on in clinic B for so and so. If you’re interested, please go and call this doctor”.

M(2): “You must call all the patients and explain to them on the phone”

M(3): “Exactly, the patient will be very upset and will want to know how you got their information. The attending physician must call the patient to ask for her consent before the researcher can recruit them.

Respondents discussed privacy concerns when the recruitment setting occurs in a public space whereby the illnesses of patients can be identified:

“ No, I would say that if there is recruitment material as in banners or something and the doctor basically put it in a public place and then the patients come for a meeting on that day and the door is open so everyone knows know there is a banner….”It's basically identifying that all those people wanted to participate....to have this condition.

“In his work, he [investigator] is at his office and he’s telling people that I’m having a meeting inviting HIV patients for the research. So, when the patients come, they are stigmatized… this is a kind of invasion to their privacy.”

Respondents mentioned the precautions that should be taken to protect privacy:

“In the event of the recruitment of participants, to maintain the privacy of patients, we should not make them wait in line like the case in clinics and so on. As a researcher, I should create a well-organized and timed schedule for every participant.”

“I would like for an investigator to recruit me… in a private place while he is taking my consent.”

Respondents mentioned a privacy issue associated with the signing of the informed consent form:

“There is worry about privacy….you started by saying there will not be any identifying information…[But a] signature is an identifying information…we cancel the signature or the first part [where one states that will not be any identifying information”

### Privacy issues during the research

Participants discussed several privacy issues that occur during the conduct of research. One issue revolves around the type of the data collected and the methods employed to collect data. In regard to type of data, one respondent mentioned the following:

“In some researches, like dermatological ones, pictures and even videos are taken. Here, the patient really starts worrying about his privacy and whether or not it will be maintained.”

Privacy issues related to gender can occurs when data are obtained during the physical exams at follow-up visits, specifically when women prefer to be examined during a clinical trial by a female doctor. One respondent said the following:

“In Saudi Arabia this is a normal practice . If I am a female, I would be examined by a female doctor. In Saudi, it's more of a concern.”

A similar gender issue arises with data collectors, individuals who are employed to distribute and collect completed surveys. One respondent said the following:

“Another popular example in Sudan [regards] …the data collector….should be one male and one female. It is impossible to send two males for example to collect data from any of [the] female in some areas.” ….This may be related to cultural issues.

One respondent brought up an issue when a third party becomes involved with translation of participants’ responses and hence, has access to the data:

“ I had two experiences, the first was a questionnaire we did in a village where most of the people were Amazigh [berber] and I don’t speak the Amazigh, we were three doctors who don’t speak it, so they brought someone to translate for us, that person was from the Douar [local community], not someone we brought with us, we had a 14 years old girl who speaks Arabic and Amazigh and she had access to all the information of the women of that Douar”

“The second involved when we were working on a questionnaire with the prisoners and for sure it was impossible to interview them without the guard standing beside you the whole time even when the patient answers or says something, the guard starts to look at him or judge him by his look, I think these conditions didn’t respect any privacy.”

Another respondent mentioned an issue with informational privacy when third parties who are not part of the research team is hired to enter the data in spreadsheets:

“Usually the people who do the research are not the same who enter them in Excel. For example, usually we have 4 or 5 persons who have no relation with the research to whom we give the documents, sometimes with the names of the participants on them, these persons will read all the information, there might be someone they know…. People gossip… [Data entry persons must be governed by specific laws, for example, the data treatment must be done in office, so they don’t take the documents with them to their homes. Or we mask the identity of the patient to be protected….”

### Observation research

Respondents discussed privacy issues in observation research in which behaviors of participants in a stressful situation were written down without identifying information and without prior informed consent. The respondents discussed the need to inform the participants after the research.

F(1): I think before anyone leaves; you should tell them I've taken notes about what happened. If you agree that I share these notes anonymously, please let me know and sign here, maybe someone doesn't want to.

F(2): If I was in the room, I would just like to know that this had taken place. I wouldn't mind. But I would just want to know.

F(3): But, no one will be identified.

F(2): But are they publishing the full conversation?

F(1): But if I was in that hospital and I was reading, would I be able to tell who person A is?

F(2): This is sort of the point I was trying to reach. If I'm sitting in this room and I was part of this rage, and then when this was published later on, I as reading it and I didn't have a clue that was me, then I think that the de-identifying was done right.

F(1): No, but still, you're using something I have done to publish, and I would want to know.

### Privacy issues after the research has concluded

Respondents raised several privacy issues involving data collected from research participants that were relevant after the conclusion of the research study.

#### Secondary research

Respondents discussed the use of data in secondary research and whether it was necessary to re-consent the research participants. There were contrasting thoughts regarding this issue and respondents offered subtle degrees of differences. In the following exchange, respondents thought that re-consenting was not necessary if there was originally “blanket” consent

M(1): “The patients did give their consent for you to use the information, so it no longer belongs to the patient.”

M(2): “But they consented to study A not study B.”

M(1) “In that case we must ask for consent for the current study and all the studies thereafter… it is because from the beginning there was no mention of a second study. So, there should in theory be a second consent request to cover the new study.”

M(3): “So, in the end it all depends on what the patient signed for, if he signed for one specific purpose, we cannot ethically use his information for another, except if he signed consent to an open study [blanket consent]”

In another exchange, several respondents discussed that re-consent was not necessary if the data/samples were de-identified.

M(1): Let's say there's no consent about using data for future uses. The data is being pulled out without any identifying information.

F(1): I wouldn't mind if it's not identifying and it's not affecting anything.

F(2): And it does not say who I am.

However, others thought that while there was not a privacy concern (i.e., no consequences), there was still a privacy violation for the participant.

F(1): No privacy concern, but it's a violation. It might not affect anything that happens. It might not affect me directly; however, it's still a violation because this person had access to my information without my permission.

Respondents discussed re-consenting issues when samples are prospectively collected for future secondary research:

F(1): There is a privacy issue in a research study when the researcher intends to create a reservoir of blood samples, and you're not saying in the informed consent that you're going to have a reservoir of blood samples that you're going to be using for X amount of time after the study has been finalized, and these are very common in numerous studies.

F(2): Yes. That if you're going to use it for wide marker sampling or whatever, and you're not telling the patient that you're going to be using it after the study has been finalized or after he's been out.

F(3): But in such case, the subject has to be reconsented again

M(1): Or you can use the same main one if they have it planned from the very beginning and he checks a box or sign to agree

F(2): Something I heard about, but I wasn't the monitor involved in this study, the consent had two check boxes, one of them for the consenting and agreeing to participate, and the other one was for the genetic testing,

#### Data sharing and security

Respondents discussed privacy issues involving sharing databases for secondary research with other investigators and the responses reveal a tension between when anonymity is sufficient and when recontact is necessary:

M(1):“…...If the patients’ identities are protected, I see no problem. For me the important thing is anonymity.”

M(2): “My morals tell me that I have to call the patient but are there laws or rules that tell you for example if you have a list of contacts or patients as it happened in the example of the department to know what you should do”?

Other responses demonstrate uncertainty regarding the data protection law in Egypt and the lack of training regarding concepts involving data security:

F(1): It's not really all that regulated yet. It's not yet regulated, and we have-- we actually have one law that is not working yet in our Constitution. We have a law--

F(2): What law?

F(1): Confidentiality of data law-- data confidentiality law. It's in the governmental Constitution, but it's not an active law. It's inactive. We have a law, it's not a draft. It's listed, but it's inactive.

F(2): It’s inactive because of the government. That's why the whole concept is not yet established of data privacy protection.

F(2): Also, because physicians are not told [about this law] while in medical school. I recall back when I was at the university, we had a training at one of the hospitals…and then in one of the clinics, the doctor had a patient and he was discussing all her medical history in front of us, although this is none of our business, none of our concern, not even of medical value, and then he started discussing her husband's medical history…one of the group talked to the doctor and said you sort of embarrassed her because you were saying too much details, and he was like, “As if she was bothered.” He was just underestimating the way she was feeling or underestimating the information related about her.

Another respondent demonstrated explicitly the need for training:

F(1): I was at one site and a researcher was literally telling me that he's doing research study about some disease and that he's basically pulling the data out of the database of the hospital and sharing it with a medical company to get the results out of it.

M(1): To get what out of it?

F(1): To do a research without even informing them. And then I was speaking to this doctor, and I was telling him, you know what? If it was me, I would sue you, and he basically told me, “No. You don't have the right to.” I'm like, “This is my information. This is my identity. This is my everything.” And he basically said no, so it happens that sometimes doctors don't get the concept of privacy and they basically put out the data from the database. If it's done for internal hospital research, it could be fine because the data is already shared in the system. However, if they are sharing it for a different entity, like for separate companies or other people outside of the same institute, it is a concern, and they don't get it.

Several respondents discussed the issue of biobanks and genetic research and the return of results. In the following exchange, the respondent favors that the return of results should be dependent of the choice of the research participant:

M(1): Well, let’s say I take pathology samples and conduct protein retention for such samples, and it turns out that such proteins actually have cancer. I have two options in that case, I can either inform the person of the results, or I can hide them from him.

F(1): Given that you actually know which patient that sample belongs to, correct?

M(1): Yes. When it comes to ethics in the field of pathology, if the research team will use material that belongs to other people, we conduct anonymization of the names. If a genetic research, rather than a Pathological one, is conducted using the same samples, here the person is free to decide whether or not he would like to know the results.

#### Group harms

In one of the focus groups, a respondent raised the issue of sharing de-identified data taken from a community or even a country and points out that there can group harms even though the data is individually de-identified:

F(1): "If you did a research, social or clinical research and at the end you find a certain group of people with a certain disease and you disclose that in a certain publication, definitely you’re going to affect that group socially, probably economically…"

M(1): It could stigmatize a population or a tribe.

F(1): But individually? Is there an individual privacy violation?

M(2): There’s group privacy. Can a village have its own privacy concerns?

F(1): I think group privacy cannot affect the direct individual, rather than the community or a country…. Like for example there’s an illness in area and a declaration of communicable disease in X area, that declaration is the responsibility of the government not everyone, because the declaration is like breaking the privacy.

M(1): Which privacy?

F(1): Privacy of the community not individual. if there is a serious illness or stigmatizing illness and if you publish that this area is having this prevalence of HIV is four times the prevalence of HIV in the rest of the country. This is what I call it group privacy, the publication of such information I think will create problem.

## Discussion

We report on an explorative qualitative study investigating the perspectives of research professionals from the Arab regarding privacy issues in research. Our major findings include: 1) respondents discussed a broad range of privacy issues that occur during the three major stages of the research process: before, during, and after the completion of research; 2) research professionals revealed their perceptions of patients’ concerns regarding assurances of privacy in the research setting compared with clinical care; and 3) respondents promoted the need for clarification of existing data protection standards as well as the need for training in practices involving the use of data in secondary research. The themes/sub-themes associated with these findings are shown in Table 2.

The respondents in this study expressed the idea that privacy meant control of access to information about oneself and their physical space. Several respondents discussed the intrinsic value of privacy as a prerequisite for individuals’ autonomy by controlling the image that they portray to the public; a concept that is similar to that of other commentators [22, 2]. Other respondents mentioned the instrumental value of privacy as enabling people to keep “disruptive material out of the public arena” and hence, shields them from the judgement/bias of others [23, 2].

The research professionals discussed a broad range of different types of privacy situations in the research setting (informational, physical, and proprietary). Our interest was not with assembling materials for an analysis of privacy, because, as noted in the Introduction section, the concept of privacy is complex and contested by lawyers, policymakers, philosophers, and other scholars. As such, we did not require our respondents to articulate a sophisticated analysis of privacy. Rather our interest was to determine what often gets described as threats to privacy and efforts to acknowledge and protect privacy in the research setting. The examples of privacy included informational, physical, and proprietary types of privacy.

In regard to privacy issues **prior to the research**, respondents brought up several circumstances that occur during recruitment efforts in which standards are lacking. Unresolved privacy issues include the methods by which potential research participants are identified and the means of initial contact. Respondents voiced a variety of opinions regarding when potential research participants are directly contacted by the research team after having accessed their medical records to determine which patients meet study criteria. Several respondents thought that researchers’ access to these records violated informational privacy. In contrast, other focus group respondents thought that such an approach does not violate privacy if an appropriate institutional affiliation existed between the prospective participants, their physicians, and the research team. These observations reveal a need to adopt common standards for data access for recruitment practices that achieve the appropriate balance between respecting privacy and enhancing participant accrual. The Health Insurance Portability & Accountability Act (HIPAA) regulations in the U.S. can serve as an example of meeting such a balance. This law permits a Privacy Officer, usually the Institution Review Board (IRB), to approve a partial waiver of consent and HIPAA privacy authorization for the purpose of identifying potential participants for research [24]. Whether Arab countries in the Middle East should adopt a similar approach requires obtaining the attitudes of the involved stakeholders.

Respondents also brought up the method of initial contact and the privacy concern that occurs when investigators approach patients in the hospital to discuss enrollment in research. Such unauthorized visits might be seen as a violation of physical privacy in the sense that patients’ physical space (sometimes referred to as a right to solitude) has been intruded. Interestingly, the methods by which initial contact is made with the potential participants are not specified in the U.S Federal guidelines and are hence guided by individual IRBs’ policies [25]. For example, several IRBs recommended that health care providers initially contact their patients to obtain their authorization to have investigators approach them for recruitment. The IRB at the University of Kentucky exemplifies this approach [26].

However, studies reveal that a variety of approaches are used in the research setting. For example, in a survey of U.S. cancer registries regarding their policies for contacting patients to participate in research, of those registries who allowed contact with their patients, 87.9% required or strongly recommended involvement of the treating physician, and a majority of the registries (82.8%) thought that such involvement only needed notification of the physician notification rather than physician permission. After the involvement of the treating physician, patients were approached more often by the investigator (60.6%) rather than by the registry staff (36.4%). Hence, the strategy used most frequently represented the least restrictive approach, which required investigators to notify patients’ physicians followed by direct investigator contact of patients [27]. In a survey of patients’ perspectives on research recruitment through cancer registries, 68% of patients said they preferred that researchers contact them directly about their interest in research participation rather than checking with their physician first and among patients who desired physician involvement, most preferred physician notification rather than a physician permission approach [28]. Further research is needed in the Middle East to examine the preferences of patients, physicians, and investigators in order to determine acceptable approaches that both protect patients’ privacy and avoid overly restrictive policies that unnecessarily impede patient accrual [27].

Finally, the setting of the informed consent process was raised as a potential physical privacy concern. For example, recruitment notices are placed in a public space asking those interested to meet in an open room or hall at a specified time. As such a meeting place typically mentions the nature of the illness being studied, the stigma that stems from such recruitment efforts can be substantial. While such a recruitment technique might be convenient for the research team, the focus groups noted that it offers no assurance of privacy. They made the point that privacy protection in research extends not just to actual participants but also to potential participants.

The respondents mentioned several privacy situations that occur **during the research.** One involved violations of informational privacy in which third parties would have access to research participants’ data. These would include non-research personnel required to help with translation between researchers and participants during focus group discussions and also data entry research personnel who would have access to personal information. Respondents discussed that Informed consent forms should mention the range of individuals who would have access to private information. Such transparency in the consent forms would give potential research participants the necessary information to decide whether to additionally waive their privacy rights.

Focus groups respondents also mentioned a concern with physical privacy that might occur when female research participants undergo physical exams during the research, as they might have preferences to be examined by a physician of the same gender. A similar issue occurs when female participants are approached by data collectors, who need to be of the same gender. This privacy issue, as noted by the respondents, tend to occur more often in the rural settings and is representative of cultural influences on the scope of privacy. These examples demonstrate the high value on modesty that exist in certain Arabic regions and while it is usually emphasized for women, it is important for both sexes [29]. Indeed, “whether or not a privacy invasion will have occurred in a case of “touching” will depend on the privacy norms found within the culture in question” [1, 30]. As such, we can appreciate how culture, which determines particular social norms based on beliefs, customs, traditions, manner of life and people’s relations with each other in a society, contributes to the importance and expectations of privacy. Indeed, notions about modesty vary between cultures, which may lead to differences in the scope of protections for physical privacy [29, 31].

In regard to privacy issues **after research is completed**, respondents mentioned an informational privacy issue regarding sharing of research participants’ data with investigators from other institutions for use in secondary research. Data sharing for further scientific research is an increasingly important topic for the pharmaceutical industry and other organizations that sponsor and conduct clinical trials [11]. Focus group respondents discussed their perspectives regarding the heightened sensitivity that many research participants might have with sharing their genetic information with third parties compared with sharing of common health information. Sharing of genetic information represents a type of proprietary privacy, insofar that genetic information relates to what constitutes a person. Several studies have shown a restrained willingness of patients to share their data with researchers from other institutions. Abou-Zeid and colleagues in their study investigating the views of Egyptian patients toward the use of blood samples for future research, 53.3% of participants were willing to share their samples to other investigators without re-contact. Corresponding results were higher for sharing of samples to other Arab countries (62.0%) compared with sharing of samples with European countries (41.8%) and with the US (37.2%) [32] . In another study involving Egyptian patients’ perspectives and attitudes towards biobanking issues, the investigators found that only 32.4% agreed to share their biospecimens with institutions abroad and only 27.8% supported such sharing with pharmaceutical companies [33]. In an Italian survey involving family members of patients attending an outpatient clinic, 86% of the participants thought it was permissible to share their biospecimens and related data with non-profit organizations, but only 30% agreed with such sharing with for-profit organizations [34]. Further studies are needed to determine the factors associated with patients’ willingness for data sharing.

The respondents debated the conditions under which data can be accessed for secondary unspecified research. They discussed re-consenting research participants for such research as well as the alternative use of broad consent that would obviate the need for re-consenting. Standards are needed in Egypt and Morocco as well as in other Arab countries specifying the conditions under which broad consent would be allowable. The use of broad consent would require strong data protection laws that regulate the collection, handling, disclosure and use of personal data as well as the privacy rights of individuals. Nearly every country in Europe and many in Latin America and the Caribbean, Asia, and Africa, have now adopted comprehensive data protection laws [35].. In 2018, the European Union’s General Data Protection Regulation went into effect [36]. Recently, there has been a trend across the Middle East to modernize data protection standards to be in alignment with international standards [37]. Egypt and Morocco recently passed data protection security laws. The Egyptian law prohibits the collection, transfer, storage, preservation, or processing of sensitive personal data unless there is written consent of the concerned person [38]. In Morocco, personal data protection is governed by Law n° 09–08 of 18 February 2009, relating to the protection of individuals with respect to the processing of personal data and has made recent efforts to align this law to that of the EU General Data Protection Regulation [39]. But, as indicated by one of our respondents from Egypt, additional efforts are needed with implementation of Egypt’s data protection law as well as further training for health care providers regarding the contents of the laws’ regulations.

The above examples demonstrate that our respondents were cognizant of a broad range of privacy concerns, although informational privacy usually dominates the discourse of privacy in research. Indeed, “there has been relatively little attention paid by philosophers to physical privacy concerns in medicine compared to informational concerns” [6]. This is unfortunate, as patients bring with them strong expectations of modesty, solitude, and bodily integrity to doctors’ offices, hospitals, and other health care settings, as well as to the research settings, as discussed by our respondents. “These expectations that they will not be needlessly touched, crowded, gawked at or imaged relate to the need for psychological comfort, dignity and security” [6].

Domination of informational privacy at the expense of other types of privacy is demonstrated in research ethics guidelines, as we previously mentioned in the Introduction. The focus on confidentiality of data after patients grant a waiver of privacy rights ignores situations that involve other types of privacy. But when patients provide informed consent to participate in research and waive some of their privacy rights they are granting researchers’ permission to access intimate aspects of their persons as well as their sensitive health information [40].

The prominence that informational privacy holds in research ethics discourse might explain why the terms “privacy” and “confidentiality” are frequently used Interchangeably. But privacy and confidentiality represent separate concepts. Such a conflation “sacrifices the important distinction that something can be private, known or felt, exclusively by an individual…but then deliberately extended into a confidential realm if it is revealed [by the patient], with restraints on use and further disclosure, to another party” [41]. Indeed, “confidentiality serves privacy” [41]. As such, informational privacy represents a right that can be violated, whereas confidentiality represents an obligation towards data security, thus ensuring informational privacy. While the relationship between confidentiality and privacy is appropriately restricted to information privacy, other types of privacy need to be considered in the research setting.

It is informative to realize that the Quran, which governs the practices of everyday life (including medical and research practices) for Muslims, provides a robust discussion of privacy [42, 43]. Islamic religious law, which is based on the “essential greatness of human beings”, recognize several human rights, one of which is the right of privacy [44]. Safeguarding and keeping of people’s “essential greatness” is dependent on the recognition and keeping of one’s privacy and when individuals keep each other’s privacy, they respect human beings’ dignity, in other words, their “essential greatness”. Islam recognizes the scope of privacy to include bodily privacy, communication and information privacy, and territorial privacy.

Several research professionals commented that patients might have a greater concern about the existence of privacy protections in research compared to the clinical setting; one respondent mentioned that patients might need “more guarantees” that privacy will be protected during research. Several studies have shown the extent of patients’ privacy concerns in the research setting. For example, in a study involving Jordanian patients, nearly 40% cited the fear of a negative impact of research on privacy as a discouraging factor towards participating in biobanking research [45]. In an Egyptian study investigating Egyptian attitudes toward biobanking, 35.9% thought that samples and data might be used in a way that would breach privacy [32]. Comparable results have been shown for patients in the U.S, as one study showed that more than 60% expressed worries about the privacy of their health information regarding participating in biobanking research [46].

The respondents remarked that patients might be more likely to waive their privacy rights for research compared with medical care if there is some compensatory benefit. In the case of clinical care, the benefit is clear — the offer of therapy and treatment. In the case of research, no equivalent offer can typically be made. Hence, potential research participants might resist access to their privacy due to a lack of direct benefit from the research. Several studies from the U.S.tend to substantiate the respondents’ claim that patients are more willing to grant permission to their privacy when there is a prospect of a benefit. For example, in one large U.S. study that investigated public opinions regarding participating in a genetic biobank, concerns about privacy were related to lower willingness to participate in a biobank. However, when respondents were told that they would receive a larger financial compensation ($200) and that they receive individual research results back, privacy concerns did not impede willingness to research participation [47]. In another study investigating the views of U.S. public towards biobanking research, willingness to participate in such research was higher if participants perceived more benefits [46]. Similar studies should be performed in the Middle East to confirm the existence of a relationship between a benefit and privacy.

Focus group respondents also expressed the concern that potential research participants’ general misunderstanding of the aims of research might also limit their participation in research. The primary aim of research is to generate knowledge to advance the health of society rather than a means to provide a direct health benefit to patients. Several of our respondents held the opinion that patients do not realize that research advances the public health of society. Accordingly, investigators need to explain to potential research participants (and also included in informed consent forms) that the benefits of research is to advance the health of society, which could serve as a justifiable reason for patients to waive their privacy rights coupled with assurances that their privacy will be protected.

Finally, the focus groups highlighted the importance of trust as another reason why potential research participants might be more hesitant to waive their informational privacy in the research setting compared with the clinical setting. Several authors cite trust as a central component of the research participant-researcher relationship [48, 49]. In a Jordanian study investigating the public views toward biobanking research, a belief in trust affected a willingness to participation positively [45]. In a large survey in the US, willingness to participate in a biobank was lower in individuals who reported lower levels of trust in the healthcare system and their researchers [46]. In low- and middle-income countries, failure to engage with communities has been recognized as a cause for erosion of trust in researchers [50].

The question of trusting the institutions supporting and monitoring the research and researchers did not arise in the focus groups. In other words, people seemed to view research participation as resting on the relationship between participants and the research team rather than resting on the more complex relationship of participants, research team, and the institutions overseeing the research study. Whether introducing more clearly the relevance of institutional support and monitoring affects people’s trust of the research team remains to be studied.

The above examples of why patients might refuse to waive their privacy rights to participate in research (e.g., lack of perceived direct benefit, misunderstanding of the aims of research, and lack of trust) need further exploratory studies with patients, as these factors affecting privacy concerns have specific implications on what changes need to be made to enhance recruitment strategies.

We realize several limitations to our study. First, this study accessed the views of respondents from only two countries in the Arab region, Egypt and Morocco, and hence might not be generalizable to other countries in the region, especially those countries with more conservative values. In a similar fashion, this study was also limited to the perspectives of investigators, clinical research associates, and members of RECs. Also, the perspectives of our respondents regarding the views of research participants regarding privacy concerns might not be equivalent to the actual perspectives of patients regarding privacy concerns in research. However, our study represents an exploratory study and hence, the respondents’ perpectives can now serve as a foundation for quantitative studies involving views of individuals involved in research and of the actual views of patients.

## Conclusions

From this exploratory study we recommend the following: 1) development of standards regarding privacy protections during recruitment efforts, especially involving access of patient’s databases and initial contact of potential research participants; 2) additional training for undergraduate, graduate, and investigators regarding implementation of their country’s data protection laws as well as elucidating best practices with data security in secondary research; 3) a quantitative study involving investigators and REC members to determine their knowledge, attitudes and practices regarding the range of privacy issues that occurs during the different stages of research; 4) a quantitative study involving patients to elicit their perspectives and attitudes regarding their potential privacy concerns in research, which can lead to appropriate recruitment efforts that protect their privacy rights.

## Supplementary information

**Additional file 1.** Interview guide for focus group discussions.

## Data Availability

The datasets used and/or analysed during the current study are available from the corresponding author on reasonable request.

## Abbreviations

CIOMS: Council for International Organizations of Medical Sciences
EU: European Union
FGDs: Focus group discussions
HIPAA: Health Insurance Portability & Accountability Act
IRB: Institution Review Board
REC: Reasearch Ethics Committes
U.S.: United States
