# Supplementary material for: Perspectives regarding privacy in clinical research among research professionals from the Arab region: an exploratory qualitative study
Source: BMC Med Ethics. 2020 Apr 15;21:27. doi: 10.1186/s12910-020-0456-9 (PMC7158072; doi:10.1186/s12910-020-0456-9)
Supplement: Supplementary file 1 — Additional file 1. Interview guide for focus group discussions. [file 12910_2020_456_MOESM1_ESM.docx]

Privacy concerns in clinical research in the Middle East and North Africa (MENA)

**Interview guide for focus group discussions**

**Investigators and Members of RECs**

1. INTERVIEWER: Have the participants discuss their concepts of privacy and its importance by raising the following scenario: “I was outside this hotel and there was signs that this place is CCTV monitored, how do you feel about that?”
2. “What does privacy mean you? What aspects of your life do you consider as being private in everyday life?

Types of Privacy Issues to look out for during the discussion

informational privacy

proprietary

association

body

solitude

bodily integrity

1. INTERVIEWER : After this response, please ask the participants about specific types of information that are considered sensitive.
2. Why is privacy important to individuals?
3. How much do people in your country value their privacy?
4. Is it different with different types of privacy?
5. Do people understand the impact of privacy?
6. What kind of harms can occur with invasion of privacy?
7. What are privacy expectations or what kind of privacy concerns are there during medical treatment?
8. What privacy concerns are there with research? AT THIS POINT, PLEASE REMIND THE PARTICIPANTS ABOUT DIFFERENT TYPES OF RESEARCH STUDIES, E.G., BLOOD SAMPLING, SURVEYS, OBSERVATIONAL, CLINICAL TRIALS.
9. Are privacy concerns in research different from medical care?
10. What privacy concerns are there before the study begins?

INTERVIEWER: Items to expect include recruitment and informed consent process.

1. What privacy concerns occur during the research?

INTERVIEWER: items to expect include follow-up visits, blood sampling, examinations.

1. What privacy concerns occur after the research?

iNTERVIEWER: YOU MAY NEED TO BRING UP PUBLISHING AGGREGATE DATA (DEIDENTIFIED), PHOTOS, ETC. ALSO, SHARING DATABASES AND BLOOD SAMPLES WITH OTHER RESEARCHERS? ALSO, BRING UP RETROSPECTIVE RESEARCH.

1. What kind of safeguards can ensure privacy in the research setting?
2. For REC members, ask what kind of privacy issues have they discussed during their meetings?
3. For investigators: what kind of privacy issues have they encountered with their research participants?
4. Of all what we have discussed, what is the most important issue in your opinion?
5. Is there anything else anyone would like to bring up?
